# Aboveground and Belowground Male Population of the Invasive Citrus Mealybug *Delottococcus aberiae* De Lotto (Hemiptera: Pseudococcidae)

**DOI:** 10.3390/insects16070651

**Published:** 2025-06-22

**Authors:** Rosa Vercher, Adrián Sánchez-Domingo, Isabel Escriche

**Affiliations:** 1 Instituto Agroforestal del Mediterráneo IAM (UPV), Escuela Técnica Superior de Ingeniería Agronómica y del Medio Natural, Universitat Politècnica de València, Camino de Vera, s/n, 46022 Valencia, Spain; adsando1@etseamn.upv.es; 2 Instituto de Ingeniería de Alimentos FoodUPV, Universitat Politècnica de València, Camino de Vera, s/n, 46022 Valencia, Spain

**Keywords:** sampling methodologies, population dynamics, natural enemies, soil distribution, male captures, Coccoidea

## Abstract

The invasive *Delottococcus aberiae* is one of the most harmful mealybugs on citrus in Spain, since it causes deformations and the premature falling of fruits in citrus orchards. To improve control strategies, this study evaluates its population above- and belowground, their distribution in the soil in addition to identifying natural enemies. The distribution in the soil of emerged males varies significantly with the distance to the tree trunks with an annual average number of 0.95 males/trap/day at 0.5 m, and ranging between 0.25 and 0.32 at 1 m, 1.5 m and 2 m. There is a consistent emergence of males throughout the year, both above- and belowground, with four distinct peaks, the first two occurring in February and April. This observation is essential to manage fruit damages that follows between March and June. Among the natural enemies detected belowground the following are highlighted: Bdellidae mites (52%), Hymenoptera parasitoids (16%) and spiders (11%). When implementing IPM strategies, emphasis should be placed on adequate soil management (crops, mulching, or the release of natural enemies) and to the appearance of first generation. We propose a belowground population sampling method as a simple and practical tool to quantify this pest’s the winter and spring populations, which remain undetectable using current methodologies.

## Simple Summary

*D. aberiae* is a new invasive pest introduced into Spain in 2009. It causes severe damage by deforming fruits, and controlling it is challenging. Our study demonstrates that, in addition to living aboveground, this pest has a permanent belowground population, previously unknown. It is distributed throughout the surface area between the rows of trees and more abundant near the trunk, and consequently this belowground population must be considered when controlling this pest. Special attention should be paid to the first generation of this insect, which develops in the soil during winter and by early spring has moved to fruit. To help monitor these early populations, the authors propose a simple and practical belowground sampling method consisting of using trays with sticky traps baited with sexual pheromone. This allows for the detection of winter and early spring populations and analyzes potential ways to reduce this population.

## 1. Introduction

On a global scale, invasive insects are a significant threat to agriculture, with severe economic implications for crop production and potential risks to food security [1]. Invasive insects can rapidly spread in new agricultural areas, primarily due to the absence of natural enemies. An example of this occurred in 2009 in Spain with the arrival of the *Delottococcus aberiae* De Lotto (Hemiptera: Pseudococcidae) [2]. This insect, responsible for fruit deformations and significant economic losses in citrus crops, originates from South Africa, where it is not considered a pest [2]. It was first detected near Valencia (eastern Spain) and continues to spread [2]. By 2023 it was present in almost all citrus-producing areas of Eastern Spain (about 200,000 ha) from Catalonia in the north to Murcia region in the south [3]. According to a recent study [3], this mealybug is expanding and will eventually affect all citrus-growing areas in Spain. Its presence has also been reported in other crops such as persimmons, medlars, pomegranates, and olive trees [4]. The damage caused by this insect makes it one of the most harmful cushion scales currently found in this country. The feeding behaviour of this species leads to severe damage to fruits, distorting their shape, producing premature falling, and/or causing a reduction in their size, making them commercially nonviable [3].

To control this pest, an Integrated Pest Management (IPM) program is being implemented, combining mass trapping (with synthetic sex pheromone lures), and biological, chemical and cultural controls. Chemical control products such as acetamiprid, paraffin oil, and spirotetramat are recommended [4]. However, this application often fails to effectively control *D. aberiae* due to their resistance to insecticides, influenced by the coexistence of different developmental stages (early life stages are the most susceptible to pesticides), its protective waxy coating and its high reproduction rate. In addition, this insect can find concealed areas within the plants thereby avoiding insecticide sprays [3,5]. Since 2020, classical biological control programs have been developed in Spain by introducing a parasitoid insect *Anagyrus aberiae* Guerrieri, from South Africa [5]. Previously, it was demonstrated that the Spanish native and naturalized parasitoids failed to control this mealybug due to parasitoids high immature mortality, encapsulation by the host, and host defensive mechanisms (evasive movements to thwart parasitoid oviposition and secreting defensive exudates) [6]. This type of control is combined with a summer release of the predator *Cryptolaemus montrouzieri* (Mulsant) with the aim of reducing populations, when mealybugs reach their peak. An important breakthrough was the discovery of the sex pheromone of *D. aberiae* [7], which is now commercially available for mass capture using the “Attract and Kill” technique (Vynyty^®^ Citrus* Bayer).

Despite the control strategies currently in place, this pest continues to account for 30–70% crop loss [3] prompting the government authorities to provide subsidies to producers as compensation [8]. Therefore, on-going research is fundamental to improve control strategies, focusing on the pest’s biology and creating fast and efficient sampling strategies to aid in decision-making.

One of the earliest studies focused on the seasonal distribution of *D. aberiae* in citrus trees was conducted by Martínez-Blay et al. [9,10]. They observed that *D. aberiae* was found mostly in the canopy, and from February to September, these mealybugs migrated to the trunk and soil. These authors, by collecting soil samples, which were bagged and transported to the laboratory showed that female and immature mealybugs move in both directions between aboveground (canopy) and belowground (soil) habitats. They located some nymphs and adult females belowground from February to September, at very low levels, and only in the 30 cm around the trunk. Given the challenges associated with soil sampling and the unavailability of an identified pheromone at the time, Martínez-Blay et al. [9,10] did not quantify the belowground population and its annual dynamics. It is also unknown how abundant *D. aberiae* is belowground compared to the aboveground population or how it is distributed in the soil

With all this in mind, the objective of this study is to quantify the presence of *D. aberiae* (males) in the soil and in the aerial part (males, females and immatures stages), its annual population dynamics and its distribution belowground, close to the tree trunk. In addition, natural enemies emerging from the soil are studied, due to its possible role as biological control agents for *D. aberiae*. Addressing these questions will improve sampling methods and allow for the implementation of new management strategies.

## 2. Materials and Methods

### 2.1. Study Area and Citrus Varieties

**Figure 1:**
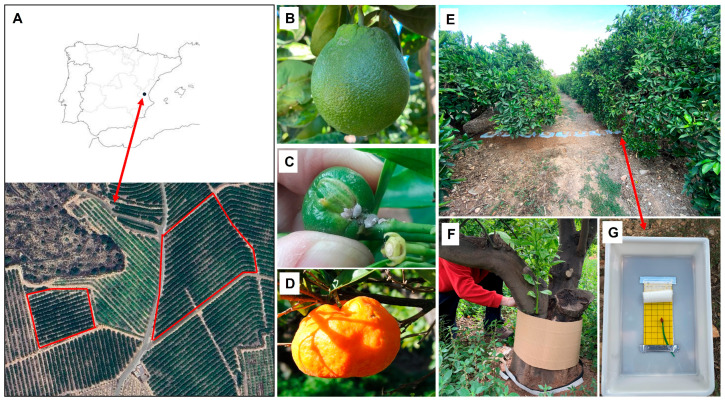
Maps showing the geographic location of the study (**A**). Damages caused by *D. aberiae* (**B**), slight fruit deformation; (**C**), aberrant fruits; (**D**), fruit with a clear loss of symmetry). Some sampling methodologies used to conduct this study were: hard plastic trays with sticky trap baited with synthetic *D. aberiae* sex pheromone for the soil (**E**,**G**) and corrugated cardboard bands on citrus trunk (**F**).

The field experiments were carried out on a citrus farm of 20 ha located in the Valencia Region (Eastern Spain, 39°64′ N 0°42′ W), the most important citrus production area in Spain. The farm belongs to the Cooperative “Sant Vicent Ferrer de Benaguasil”, which is responsible for the management of these fields. The use of pesticides is minimized since it is under agroecological transition. The farm is divided into sections to facilitate irrigation, cultivating different varieties of citrus (5 to 8-year-old trees). All sampling was conducted on two Lanetale variety citrus orchards of 0.6 ha and 1.8 ha (Figure 1A).

The mealybug *D. aberiae* invaded the farm in 2020, rapidly spreading, displacing previously existing mealybugs and becoming the dominate pest. The pest management strategy (which started in 2023 and continued in 2024) was carried out following the principles of IPM, combining the use of pheromone “attract and kill” (Vynyty^®^ Citrus* BAYER), with one treatment with Acetamiprid in April and inoculative releases of *D. aberiae* natural enemies in July (C. *montrouzieri* and *A. aberiae*).

### 2.2. Above- and Belowground Sampling of D. aberiae and Natural Enemies

To sample the belowground population, the method developed by Vercher et al. [11] for *Planococcus citri* (Risso) in persimmon was employed. This involved placing hard plastic trays (50 × 30 × 10 cm, 0.15 m^2^) upside down on the soil surface after removing leaf litter and grass. A 10 × 25 cm yellow sticky plastic trap (Econex, Murcia, Spain) was placed on the upper interior surface of each tray and baited with a red rubber septum loaded with 250 μg of synthetic *D. aberiae* sex pheromone (Zentinel^®^ DAB, EPA SL, Carlet, Spain) to capture males emerging from the ground (Figure 1E,G). Following the manufacturer recommendation, the septa were replaced every 2 months to improve efficacy. Plastic trays were arranged side by side on the ground, forming a continuous line of eight trays that span the gap between two opposite trees in adjacent rows. To prevent insect intrusion from the sides of the plastic trays, loose soil was used to compact and seal the borders (Figure 1E). The experiment was conducted in the central part of the orchard and was replicated in the two other orchards of the farm (Figure 1A). Therefore, for each repetition there were eight trays, with two trays placed at each of the following the distances: 0.5 m, 1 m, 1.5 m and 2 m, from the tree (Figure 1E). In total, 25 samples with four repetitions of each of the four indicated distances were performed, with a total of 396 traps (since four traps were misplaced). To monitor aboveground populations, Delta traps (Econex, Murcia, Spain) with the same pheromone and the same yellow sticky plastic were used on each orchard. They were placed in the canopy of the trees adjacent to the plastic trays, in the inner branches (at 150–190 cm above the ground) representing 62 traps in total. From July 2023 until June 2024, above- and belowground traps were collected and replaced every 14 days.

The specimens of *D. aberiae* captured from above-and belowground) as well as the natural enemies (captured from belowground) were collected in the field and were identified and counted in the laboratory using a stereomicroscope (Nikon SMZ745, Tokyo, Japan). This task was carried out by experts from the Mediterranean Agroforestry Institute (IAM-UPV). Natural enemies were identified down to the genus or family level, depending on the type of arthropod. In some cases, due to poor arthropods conditions it was not possible to identify them to the genus or family level, and were therefore recorded as unidentified.

Previous research had shown that the most effective approach to monitoring *D aberiae* population in March and April is by capturing females and immatures using corrugated cardboard bands on the trunk [9,10]. Females and immature instars take refuge and lay their eggs or make cocoons on this corrugated cardboard bands [12]. For this reason, to evaluate the population of *D. aberiae* between March and April 2024 corrugated cardboard bands were placed around tree trunks. Four trees from two different orchards were selected. In the four trees of each orchard, the corrugated cardboard boxes were placed. Two of these trees already had the Delta trap and other two were randomly selected from the central area of the orchard The cardboard bands (approximately 40 cm wide each, Figure 1F) were wrapped around the trunks of the trees and replaced every 14 days. In the laboratory, the corrugated cardboard boxes were opened, and the adult females and immature individuals were counted.

### 2.3. D. aberiae Population on Fruits

During the period of fruit set and development in citrus, like other mealybugs, *D. aberiae* tends to aggregate and concentrate on fruits. Therefore, the most appropriate sampling method for assessing its aerial females and immatures population (aboveground) is the direct evaluation on fruits [9,10]. Fortnightly, from July 2023 to June 2024, ten trees were randomly selected from the central area of each orchard for each sampling event. From each tree, six fruits were collected from five different canopy positions: one each from the North, East, South, and West sides, and two from the centre. In total, three repetitions were carried out, two in the larger orchard (1.8 ha), and one in the smaller (0.6 ha). Therefore, a total of 180 fruits were picked fortnightly (60 fruits per replication), and examined in the laboratory under the stereomicroscope (this pest prefers to shelter within the fruit calyx) [9]. The number of fruits with presence of females and immatures of *D. aberiae* was counted.

### 2.4. Fruit Damage Caused by D. aberiae

To assess the damage caused by *D. aberiae* on the fruits, observations were conducted at the end of their growing season (October 2023 and 2024) since most damage is caused to the fruit between April and June; and it is not fully visible until the fruits have completely developed [3]. A visual observation of fruits in both orchards was carried out. To this end, twenty fruits per tree were inspected—four from each orientation (North, East, South, West) and four from the central canopy section—across a total of 23 previously randomly selected trees (13 trees in the larger orchard, and 10 in the smaller). Therefore, 460 fruits were inspected each year and categorized into four groups according to Gavara et al. [3]: 0—healthy fruit; 1—slight fruit deformation; 2—fruit deformation with a clear loss of symmetry; and 3—aberrant fruit.

### 2.5. Data Analysis

To standardise the results, the number of *D. aberiae* males captured in all traps (at the tree canopy and ground levels) was divided by the number of days each trap remained in the field (to obtain the average number of mealybugs captured daily). To calculate the catches per unit of ground level sampled area, the males per m^2^ were recorded considering that each trap at ground level covered an area of 0.15 m^2^. In the case of natural enemies, the average was estimated as number of arthropods/trap/week.

The Statgraphics Centurion XIX.64 package (Fisher LSD; α = 0.05) was used to evaluate: 1. The effect of the distance (from the trunk) on the variable “number of males captured/day” through a one-way-ANOVA; 2. The effect of the month on the above-ground and belowground male population and on the percentage of fruits with *D. aberiae* through a one-way-ANOVA; 3. The effect of month and the distance (from the trunk) on the abundance of natural enemies (arthropods/trap/week) by means of a multifactor ANOVA. Data on percentages were expressed as arcsine square-root-transformed to stabilise the variance before the ANOVAs. In the case of the daily or weekly number of arthropods per trap, the data was log-transformed (ln [captures + 1]) when necessary, to normalise residual data distribution and to homogenize the variance.

The percentage of fruit damage was quantified during two consecutive years (2023 and 2024), using a paired comparative analysis. For each level of damage, the distribution of data was first assessed for normality using the Shapiro–Wilk test, applied separately to the 2023 and 2024 datasets. Given that several groups showed non-normal distributions (*p* < 0.05), the Wilcoxon signed-rank test, a non-parametric alternative to the paired *t*-test, was used to determine significant differences between years.

## 3. Results

### 3.1. Soil Distribution of D. aberiae Males

**Figure 2:**
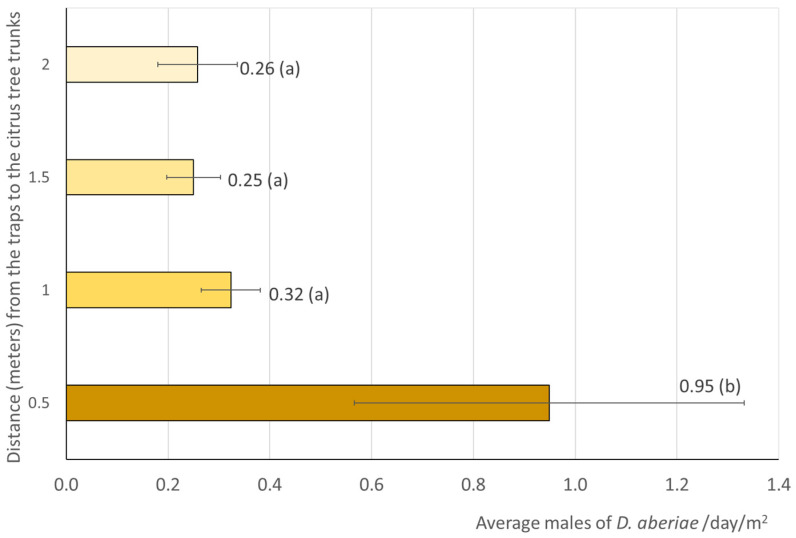
Captures of *D. aberiae* males in pheromone traps placed in trays on the ground at different distances (0.5, 1, 1.5, and 2 m) from the citrus tree trunks, in two citrus orchards (Valencia, Eastern Spain). From July 2023 to June 2024, sampling was performed at fortnightly intervals with four replications per sampling event. Data are expressed as average per day (*n* = 394) and standard error (SE). Values followed by the same letter do not differ significantly (Fisher LSD test, *p* < 0.05).

The distribution of emerged males from the soil (Figure 2) showed that the annual mean number of males was 0.95/trap/day in trays located next to the trunk at 0.5 m. This value was signifcantly higher (F_3,393_ = 2.91, *p* = 0.034) than those observed at the other distances (1 m, 1.5 m and 2 m). These last distances had the mean number of males three and four times lower than at 0.5 m, with values ranging 0.25–0.32/trap/day.

### 3.2. Above- and Belowground D. aberiae Seasonal Evaluation

**Figure 3:**
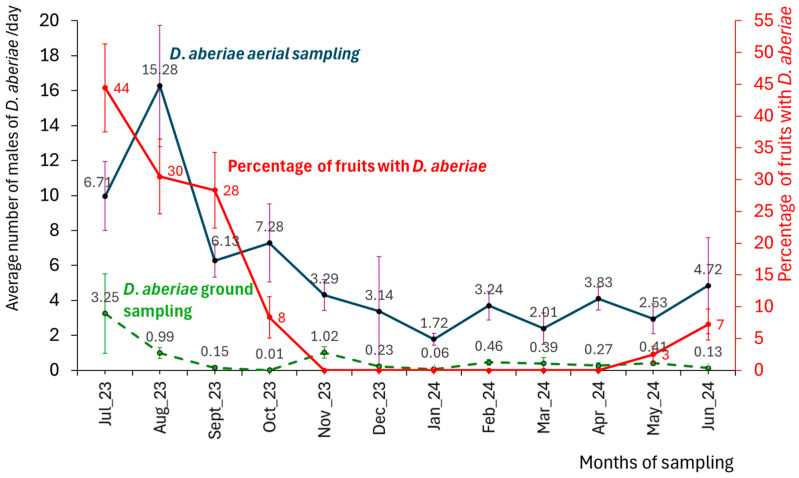
Captures of *D. aberiae* males in pheromone traps placed on the branches aboveground sampling, on belowground sampling, and percentages of fruits with females and immatures, in a citrus orchard in Valencia region (Eastern Spain). Sampling was carried out every 14 days from July 2023 to June 2024. Data are expressed as average per day and standard error (SE).

The annual average of *D. aberiae* males was 5.5 ± 0.73 males/traps/day in the aerial traps and 0.4 ± 0.10 males/traps/day in the ground traps (considering all distances). In Figure 3, aboveground male captures are compared with those from belowground, using only data from the trays located closest to the tree (0.5 m), since this is the similar distance (to the center of the trunk) at which the aerial trap was placed. It is observed that there is a consistent emergence throughout the year, both aboveground and belowground. The population peak in the ground traps occurs in July (3.25 ± 2.28 males/traps/day), with no significant differences between months (F_11,99_ = 0.95; *p* = 0.497). The lowest male counts on the tree canopy (1.72 ± 0.35 males/traps/day) occurred in January, while on the ground ocurred in October (0.01 ± 0.008 males/traps/day). Expressing the number of emerging males per day and per square meter, 21.67 males/day/m^2^ were recorded in July belowground at 0.5 m from the trunk. In the canopy traps, a significant effect of the month was observed with mealybug captures significantly (F_11,50_ = 3.18; *p* = 0.025) higher in August (mean value of 15.28 males/traps/day), compared to the other months.

When analysing the population in fruits, expressed as the percentage of fruits with *D. aberiae* inmatures and adult females stages (Figure 3), important differences were observed between months, with July, August, and September being significantly higher (F_6,26_ = 13.60; *p* = 0.000) in comparison to the other months. The maximum values were in July (44%) and the minimum in November to April (0%). In the spring of 2024, the population increases starting in May, reaching 7% of fruits infestation by June. Additionally, with the cardboard method, the average number of females and immature males observed was the same in March and April, with a value of 0.14 ± 0.04 mealybugs/day.

### 3.3. Male Distribution of D. aberiae over the Year

**Figure 4:**
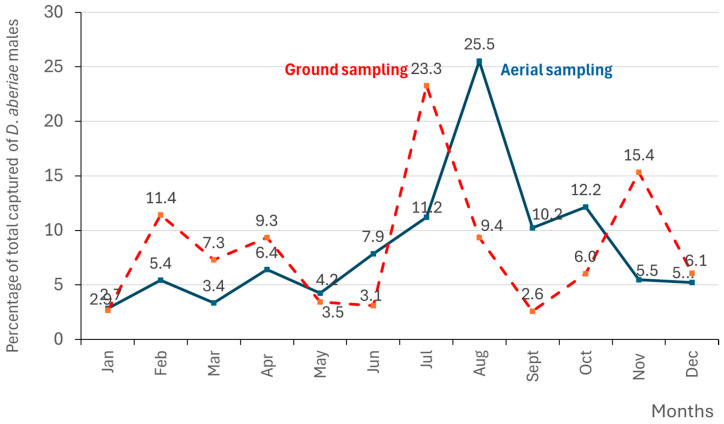
Captures of *D. aberiae* males in pheromone traps placed on the branches aboveground sampling andbelowground sampling in a citrus orchard in Valencia region (Eastern Spain). Sampling was carried out every 14 days from July 2023 to June 2024. Data are expressed as percentage of monthly catches with respect to the annual total.

For a better visualization of the number of annual generations of *D. aberiae* males throughout the months, Figure 4 represents their distribution (expressed as the average percentage at all distances) in each month, relative to the annual total captures belowground and aboveground. Four population peaks can be observed both belowground and aboveground, which could be related to four generations of these mealybugs. Belowground, they occur in February (11.4% of total of total of captures of males), in April (9.3%), in July (23.3%), and in November (15.4%). Aboveground, they occur in February (5.4% of total captures of males), in April (6.4%), in August (25.5%), and in October (12.2%).

### 3.4. Fruit Damage Level

The percentage of fruit with damage obtained at the end of the season 2023 and 2024 is shown in Table 1. The overall results indicate that in 2024 there was a higher proportion of healthy fruits (55.78 ± 4.21) compared to 2023 (31.75 ± 5.38). Likewise, in 2024 the incidence of fruits with level 1 (slight fruit deformation) and level 2 (deformation with clear loss of symmetry) was significantly lower than in 2023. The average values recorded for these levels were 40.31 ± 4.50 and 1.48 ± 0.48 in 2024, compared to 57.92 ± 4.70 and 8.92 ± 2.30 in 2023. However, for aberrant fruits (level 3), non significant differences were observed between the 2 years with percentages of 1.42 ± 0.61 in 2023 and 2.42 ± 1.11 in 2024.

### 3.5. Presence and Abundance of Belowground Natural Enemies

The total number of natural enemies identified in the yellow sticky traps on the ground, in function of the distance from the trunk are shown in Table 2. The results reveal that the most common arthropods are from the class Arachnida (70%), compared to 30% represented by the class Insecta. Bdellidae mites represent 52%, spiders 11%, and Pseudoscorpionida 6% of the total arthropods. Among the insects, the most abundant are Hymenoptera parasitoids (16% of the total arthropods), followed by Diptera (11%) and the Staphylinidae beetles (3%). Among the Hymenoptera parasitoids, the families Mymaridae, Ceraphronidae, and Scelionidae are the most common, representing 25%, 16%, and 13% of the total parasitoids, respectively. The superfamily Ichneumonoidea was also important, representing 20% of the total parasitoids, but only a few specimens could be classified to the family level, as their wings could not be clearly distinguished. Among the Diptera, the vast majority was Cecidomyiidae. This group has not been identified at the species level and, while classified as natural enemies, may be saprophagous or herbivorous.

When observing their abundance based on the distance from the tree, a gradient was shown for some arthropods, although these differences were not significant (Table 3). For example, Bdellidae and Cecidomyiidae were more abundant in the area farthest from the tree, while Pseudoscorpionida were more abundant near the tree. In other groups, such as Hymenoptera and Staphylinidae, no gradient was detected.

The multifactor ANOVA (Table 3) of the most abundant arthropods mentioned before (arthropods/week/trap), indicates that there was a significant month effect for Staphylinidae and *Alaptus* spp., wich were most abundant in September; Bdellidae, in May and June; and Pseudoscorpionida, in June. The interaction (month × distance) was not significant in any case.

## 4. Discussion

Our findings provide the first clear proof that a stable, year-round population of *D. aberiae* continuously emerges from belowground, not only near the tree (where the concentration is highest), but also across the entire cultivation area between tree rows. This finding is significant since it reveals a consistent pest population in the soil which has never been controlled. This may explain the considerable difficulty in managing this pest, which continues to cause severe damage despite the implementation of various management strategies. Previous studies using different methodologies also indicated the presence of this pest in the soil, locating nymphs and adult females belowground from February to September, although at very low levels, and only in the area near the trunk [10].

Since the same traps and pheromones were used above- and belowground in this study, comparisons can be made. However, it should be noted that the ground traps cover an area of 0.15 m^2^, while the aerial traps influence a much larger area. Therefore, some of the insects captured in the tree canopy may come from the emerging underground population. It should also be considered that the moisture that accumulates over time in the soil traps can reduce capture ability and the sex pheromone can likely disperse differently in soil versus air. Even so, these belowground captures at 0.5 m represent up to 33% of the total (above- and belowground) in July and 24% in November. Expressing the number of emerging males per day and per square meter, 21.67 males/day/m^2^ emerged in July belowground at 0.5 m from the trunk and 6.4 males/day/m^2^ considering all the distances observed in this study. Assuming that the pest is uniformly distributed throughout the orchard, 64,000 males/ha could emerge from the soil in July. This could result in an approximate total population of 128,000 adult/ha, considering that the sex ratio of mealybugs is usually 1:1 [13,14]. These findings further highlight the importance of the belowground *D. aberiae* population in overall pest dynamics.

It is possible to capture *D. aberiae* at distances greater than 3 m from the synthetic sex pheromone lures (V. Navarro, *pers. comm., 2025*). Taking this into account (but being conservative and considering that each trap attracts males located within a 3 m radius), it could be estimated that each aerial trap baited with sex pheromone attracts males within a surface area of 28.26 m^2^ (area of the 3 m radius circle). If these surfaces are included, the ground population becomes substantially more important, increasing its share of total captures from 10% to 90%. While these results are approximate, future specific studies are needed to more accurately compare these populations.

The present study identifies four populations per year both above- and belowground. Belowground peaks occur in February, April, July, and November. Aboveground, the first two peaks occur in February and April; however, the third and fourth peaks occur in August and October, respectively. These variations may be due to temperature differences in both the canopy and soil. Soil temperatures remain more stable and do not experience the wide fluctuations seen aboveground, nor do they reach the thermal peaks typical of the Mediterranean during the warmer months [15].

To date, the number of generations of *D. aberiae* has not been determined in Spain since there is a mix of developmental stages starting in the summer [9]. It is common not to have a clear understanding of the generations of mealybugs in the Mediterranean, as many species show a similar pattern, such as *P. citri* and *Pseudococcus viburni* Signoret [11]. In the study conducted in Spain by Martínez-Blay et al. [9,10], only regarding aboveground females and immatures, two generations are clearly marked, the first in spring and the second in summer, coinciding with the second and third generations observed in the present work. These authors highlighted at least three more generations: one between January and February, another between August and October, and one more between October and December. Our results, based on male population, coincide with the February and October generations, but no population peak is observed between August and October. Accurately identifying the timing of generation peaks is essential for optimising pest management during the most vulnerable stages, especially for mealybugs, since adults are pesticide-resistant and only early stages are susceptible [16]. Since most fruit deformities occur between March and June [9,10], when aboveground populations are very low, controlling the first and second belowground generation (21% of the total annual belowground males) may be the key to improving pest management and therefore, can be crucial for reducing fruit damage. Gavara et al. [3] already reported that economic losses produced by *D. aberie* were related to the population levels in spring (April–June), not to the maximum annual male catches.

In general, aboveground mealybugs are difficult to detect during winter or early in the season due to the cryptic behaviour, and clumped distribution [17]. This is the case with *D. aberiae*, which is particularly hard to locate using traditional sampling methods (aboveground visual inspections searching for individuals or sooty mold resulting from honeydew residues) during the winter and spring [9,10]. Therefore, we recommend the method utilized in this study stated by Vercher et al. [11], which focuses on the 0.5 m area surrounding the trunk, together with pheromone traps placed in the canopy as a quick and simple way to sample the initial annual populations, which could be responsible for the most significant fruit damage. Since economic thresholds for this pest are still unknown, this method provides a valuable tool for future development [3]. Although this above-and belowground behaviour is observed for the first time in *D. aberiae*, it has already been described in other species of Pseudococcidae [18]. Comparable results were obtained by studying emerging males from the soil of *Planococcus citri* in persimmons [11] and citrus (unpublished data). Also, *Planococcus ficus* (Signoret), the vine mealybug, feeds on the vine roots, trunk, cordon, canes, leaves, and fruit [19]. *Planococcus* sp., *P. citri*, and *Planococcus lilacinus* (Cockerell) infest the roots and basal region of the stem of black pepper vines damaging the aerial parts of the plant such as the tender shoots, leaves, and berries [20]. Xu et al. [21] indicate that the mealybugs *Saccharicoccus sacchari* (Cockerell) and *Heliococcus summervillei* (Brookes) feed on sugarcane and leaves aboveground and on roots belowground. There are also some mealybugs that live exclusively belowground and feed on sucking sap from plant rootlets, such as the family Rhizoecidae, previously included in Pseudococcidae, and recently separated [22].

Soil and litter fauna play a primary role in ecosystem functioning, yet their biodiversity and ecological relationships remain largely unknown [23]. In this study, we have identified the predators and parasitoids captured in soil traps. Most were predators, including arachnids (Bdellidae mites, pseudoscorpions, and spiders), as well as Cecidomyiidae and Staphylinidae, all common inhabitants of soils [24]. Many of these predators are generalists and can feed on mealybugs. Bdellidae prey on small arthropods such as soft-bodied insects, collembolans, and mites [25]. Cecidomyiidae are some of the most common natural enemies associated with mealybugs [26], such as *Diadiplosis saccharum*, which is described as a predator of *S. sacchari*, which also lives in the roots [27].

Among the parasitoid hymenopterans (16% of total arthropods), the most common are Myrmaridae, Scelionidae, and Ceraphronidae. Scelionids are relevant for being egg parasitoids of arthropods and are observed in the soil [28]. They are frequently captured in traps associated with soil and litter, and their host range is extensive, including Hemiptera, especially aphids and mealybugs [29]. All Myrmaridae are internal parasitoids of insect eggs and prefer hosts whose eggs are concealed within plant tissues, under bark, and in soil [30]. Most recorded hosts are Sternorrhyncha (Hemiptera), but the eggs of other Hemiptera, Coleoptera, and Psocoptera are also commonly attacked [31]. We also found some parasitoids from other families of Chalcidoidea, but in small numbers. Other studies report comparable results using different sampling methodologies for example, Martínez-Falcón et al. [24], researching soil and litter, identified parasitoids of Eulophidae, Mymaridae, and Pteromalidae, representing only 0.5% of the total collected fauna. Lotfalizadeh et al. [32] also identified several Chalcidoidea in the soil, however, the ecological role of this Superfamily plays in the soil is unknown. It is possible that some of these natural enemies feed on *D. citri* in the soil, but more specific studies are needed to understand the impact and role of this soil entomofauna in the control of mealybugs.

To manage *D. aberiae* soil population, we propose biological control strategies through conservation (cover crops or mulching) and enhancement (soil arthropods and entomopathogens). Agroecological practices such as the implementation of cover crops are known to contribute to the maintenance of local biodiversity in farming systems [33] and increase natural enemies in the agroecosystem [34]. Several authors indicate that a rich cover crop increases the biodiversity of ground-dwelling arthropods. De Pedro et al. [28] demonstrate that in pear orchards, the ground cover increases the abundance of several families of spiders, beetles (Carabidae, Staphylinidae), and hymenopterans (Scelionidae). Sommaggio et al. [35] found a significantly higher activity and density of staphylinids and carabids on the soil surface of a vineyard with cover crops, compared to the control (without cover crops). In a study carried out by our team (unpublished data), we found that the captures of male *D. aberiae* on citrus trees were significantly lower when there was vegetative cover beneath the trees and between rows compared to when there was only vegetation between rows. Also, soil predatory mites in citrus could be enhanced in conservation biological control by adding compost [36] or through the application of mulch [37].

As an augmentative strategy, soil predatory mites can be used to reduce soil mealybugs population, since according to Pérez-Rodríguez et al. [38] they are potential biological control agents of *D. aberiae*. In fact, *Gaeolaelaps aculeifer* (Canestrini) and *Stratiolaelaps miles* (Berlese) have been successfully released for the augmentative biological control of different pests [39].

Regarding entomopathogens, currently there is interest in using entomopathogenic nematodes (EPNs) in soil within an IPM scheme to reduce usage chemical pesticides, since EPNs is generally more suited to control soil-dwelling insect stages than aboveground insect pests [40]. Le Vieux and Malan [41] showed that EPNs, and specifically *Steinernema yirgalemense* Nguyen, et al. (Rhabditida: Steinernematidae) show great potential as biological control agents for the control of *P. ficus* soil populations. Another alternative is the use of entomopathogenic fungi (EPF) which offers several advantages, such as specificity against pests and minimal environmental impact, no risk to mammalian health and low potential for resistance development [42]. The species most often isolated from soils in temperate regions belong to the genera *Beauveria*, *Isaria* (Cordycipitaceae) and *Metarhizium* (Clavicipitaceae) [43]. They have been shown to be effective in controlling aboveground mealybugs such as *Paracoccus marginatus* (Williams and Granara de Willink) [44] and *P. viburni* [45] Soil application of EPFs in field or semi-field conditions has successfully reduced the population of pests in their soil-dwelling stage, such as the beetles, thrips, mites and lepidopteran larvae [46]. However, the impact of these agents on *D. aberiae* remains unknown, and further research is required to assess their efficacy.

## 5. Conclusions

The present study provides quantifiable evidence of an existing and persistent population of *D. aberiae* belowground in citrus orchards, primarily concentrated within the first 50 cm around the trunk and distributed throughout the soil between tree rows. This belowground population can be widespread and should be considered when implementing IPM strategies. Further studies should evaluate the effectiveness of cover crops, mulching, or the increased releases of soil arthropods or entomopathogens to reduce the severe fruit damage caused by this invasive pest. This study suggests that *D. aberiae* exhibits four population peaks annually, occurring both aboveground and belowground, which could be related to four generations of this mealybug. Notably, managing the first generation, which develops belowground in winter and subsequently attack the fruits in March and April, is emphasized. To this end, we propose our belowground population sampling method as a simple and effective tool for detecting and quantifying the winter and spring populations of this pest, which are otherwise undetectable using conventional sampling methods.

## Figures and Tables

**Table 1:** Percentage of damaged fruits at the end of the years 2023 and 2024 considering different levels: (0—healthy fruit; 1—slight fruit deformation; 2—fruit deformation with a clear loss of asymmetry; and 3—aberrant fruits). All values are reported as means ± standard errors (SE). Normality the Shapiro-Wilk test and Wilcoxon signed-rank test were applied. Statistical significance was determined at the 0.05 level: (* = significant differences and ns = non-significant differences).

	**Fruit Damage Level (%)**			
**Year**	**0**	**1**	**2**	**3**
**2023**	31.75 ± 5.38	57.92 ± 4.70	8.92 ± 2.30	1.42 ± 0.61
**2024**	55.78 ± 4.21	40.31 ± 4.50	1.48 ± 0.48	2.42 ± 1.11
**Shapiro *p* (2023)**	0.049	0.623	0.003	0.000
**Shapiro *p* (2024)**	0.151	0.028	0.000	0.000
**Wilcoxon** **signed-rank test**	W = 22.5 *p* = 0.007 *	W = 36.5 *p* = 0.003 *	W = 5.0 *p* = 0.005 *	W = 19 *p* = 0.68 ^ns^

**Table 2:** Total number of natural enemies identified in soil traps in relation to the distance to the trunk (0.5 m, 1 m, 1.5 m and 2 m). From July 2023 to June 2024, sampling was performed at 14 intervals with four replications per sampling event, in a citrus orchard in Valencia (Eastern Spain).

	Arthropods			Distance to Trunk (m)				
**Class and Order**	**Superfamily**	**Family**	**Genus**	**0.5**	**1**	**1.5**	**2**	**Total**
**Insecta**				46	42	36	49	**173**
Diptera				8	12	14	30	**64**
	Sciaroidea	Cecidomyiidae		6	12	14	29	**61**
	Empidoidea	Hybotidae	*Platypalpus*	2	0	0	1	**3**
Coleoptera	Staphylinoidea	Staphylinidae		5	2	5	4	**16**
Hymenoptera				33	28	17	15	**93**
	Ceraphronoidea	Ceraphronidae		4	5	3	3	**15**
		Megaspilidae		0	0	1	0	**1**
	Chalcidoidea	Encyrtidae		1	0	0	0	**1**
		Eulophidae		1	0	0	0	**1**
		Mymaridae	*Alaptus*	4	5	3	5	**17**
			*Camptoptera*	0	0	1	0	**1**
			Others	3	1	1	0	**5**
		Others		3	1	0	1	**5**
	Cynipoidea	Cynipidae		5	1	0	0	**6**
	Ichneumonoidea	Ichneumonidae		3	0	2	1	**6**
		unidentiffied		0	10	3	2	**15**
	Platygastroidea	Scelionidae		5	3	1	3	**12**
	Others			4	2	2	0	**8**
**Arachnida**				91	88	72	150	**401**
Araneae				13	17	15	20	**65**
Trombidiformes	Bdelloidea	Bdellidae		66	57	54	124	**301**
Pseudoscorpionida				12	14	3	6	**35**

**Table 3:** Mean and Standrad Error of the most abundant arthropods/natural enemies (captures/week/trap) identified in soil traps. Multifactor ANOVA (months and distance to the trunk) (Fisher LSD test *p* < 0.05: ns = non-significant differences and * = significant differences).

Order	Superfamily	Family	Genus	Anova (F Ratio; *p* Value)		
				**Month (M)**	**Distance (D)**	**M × D**
Diptera	Sciaroidea	Cecidomyiidae		0.94 (0.503) ^ns^	0.90 (0.441) ^ns^	1.22 (0.193) ^ns^
Coleoptera	Staphylinoidea	Staphylinidae		6.05 (0.000) *	0.47 (0.701) ^ns^	0.82 (0.758) ^ns^
Hymenoptera	Ceraphronoidea	Ceraphronidae		1.53 (0.119) ^ns^	0.14 (0.937) ^ns^	-
		Mymaridae	*Alaptus*	2.28 (0.011) *	0.13 (0.944) ^ns^	0.28 (1.00) ^ns^
	Platygastroidea	Scelionidae		0.98 (0.463) ^ns^	0.37 (0.774) ^ns^	0.79 (0.792) ^ns^
Trombidiformes	Bdelloidea	Bdellidae		13.97 (0.000) *	2.08 (0.103) ^ns^	1.00 (0.474) ^ns^
Pseudoscorpionida				7.62 (0.000) *	0.82 (0.482) ^ns^	0.52 (0.989) ^ns^

## Data Availability

The original data presented in the study are openly available in [RIUNET] at DOI: 10.4995/Dataset/10251/221324.

## Abbreviations

The following abbreviations are used in this manuscript:

EPF	Entomopathogenic fungi
EPN	Entomopathogenic nematodes
IPM	Integrated Pest-Management
